# Evaluation of the effects of silk and polyethylene terephthalate sutures on postoperative complications in impacted lower third molar surgery

**DOI:** 10.1007/s10856-023-06756-w

**Published:** 2023-10-16

**Authors:** Orhan Zeynep Dilan, Ciğerim Levent, Kaplan Volkan, Güzel Mehmet, Galayene Abdurrahman, Alsmadi Mohammad, Özyurt Anıl

**Affiliations:** 1 Faculty of Dentistry, Department of Oral and Maxillofacial Surgery, Van Yüzüncü Yıl University, Van, Türkiye; 2 Faculty of Dentistry, Department of Oral and Maxillofacial Surgery, Tekirdağ Namık Kemal University, Tekirdağ, Türkiye; 3 Faculty of Dentistry, Department of Oral and Maxillofacial Surgery, Dokuz Eylül University, İzmir, Türkiye

## Abstract

The aim of this study was to compare the efficacy of silk and Polyethylene Terephthalate (PET) sutures on postoperative complications in impacted lower third molar surgery. This prospective, randomized, split-mouth, double-blind clinical study was performed between January 2021 and June 2022 at the Faculty of Dentistry, Department of Oral and Maxillofacial Surgery, Van Yüzüncü Yıl University. The patients were categorized into two groups in terms of using suture material. PET suture was used in Group 1, and the silk suture in Group 2 for wound closure following impacted lower third molar surgery. The statistical significance level was accepted as *p* < 0.05 in the study. Forty patients (21 women, 19 men; mean age: 26.1 ± 7.25 years) were included in the study. When values for swelling and trismus evaluation were analyzed, there was no significant intergroup difference (*p* > 0.05). However, the VAS values of the silk group patients were higher at the 12th and 24th hours (*p* < 0.05). In addition, the plaque accumulation value in the silk group was higher than that in the PET group on the second postoperative day (*p* < 0.05). The results indicated that the plaque accumulation in the PET suture was less on the second postoperative day. Also, PET suture group patients felt less pain during the 12th and 24th hours. These results support to use PET sutures in impacted lower third molar surgery.

**Graphical Abstract:**

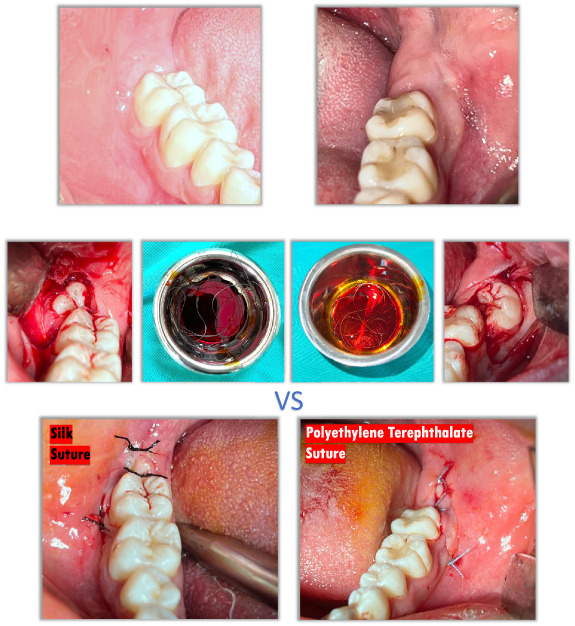

## Introduction

Third molars are the most commonly impacted teeth, and their extraction is one of the most frequently performed operations in oral surgery [1]. Complications may occur intra- or postoperatively, and pain, swelling, and trismus are the most common complications [2]. These complications are the most intense during the first postoperative 48 h, and they subsequently decrease in intensity and regress within 7 days. Complications as a result of surgical trauma are a consequence of inflammation. The general goal of postoperative treatments isto prevent or minimize complications that reduce patients’ quality of life [3].

In the surgery of the impacted third molar, the area is left for primary or secondary wound healing following tooth extraction [4]. After the teeth extraction with complete mucosal retention, the area is sutured for hemostasis and restoration of the wound edges to their original position [5]. Suture materials support the soft tissue and bring the wound edges together [6]. Thus, sutures hold the wound edges together until the edges are strong enough to resist tensile forces [7]. There are numerous varieties of suture materials with different properties [8]. Sutures are mainly classified as natural/synthetic, absorbable/non-absorbable, and monofilament/multifilament. Silk suture, routinely used in oral surgical procedures for many years, is a non-absorbable, braided suture made of natural protein filaments obtained from silkworm larvae. Owing to its braided structure, it is susceptible to bacterial infestation and is subject to progressive degradation, resulting in loss of tensile strength [7]. Despite its disadvantages, it is preferred because of its low cost and easy manipulation. In general, silk and other non-absorbable multifilament sutures have high knot security; however, owing to their surface structure, they create a retention area for microorganisms and cause an increased inflammatory reaction in the surgical site due to structural deterioration [9].

Polyester sutures show a lower tissue reaction than natural sutures owing to their synthetic structure. They can be monofilament or multifilament [10]. Polyethylene terephthalate (PET) is a polyester suture with a multifilament structure, and its advantages include biocompatibility, high homogeneity, mechanical strength, and resistance to chemical abrasion. However, the most critical disadvantage is that its surface is prone to bacterial contamination [11]. Therefore, antibacterial surface coatings are applied to prevent contamination. Local conditions in the surgical area directly affect postoperative complications in the oral region. The fact that the surgical site is in constant contact with saliva and nutrients reveals the importance of the choice of suture material, and the surgical site’s condition should be considered when selecting the suture material [9]. The PET suture may be thought to positively affect postoperative complications due to advantages related to its structural properties. The aim of this study was to compare the efficacy of silk and PET sutures on postoperative complications in impacted third molar surgery.

## Materials and methods

This prospective, randomized, split-mouth, double-blind clinical study was performed between January 2021 and June 2022 at Van Yüzüncü Yıl University Faculty of Dentistry, Department of Oral and Maxillofacial Surgery. The study was approved by Van Yüzüncü Yıl University Clinical Research Ethics Committee (Approval Number: 21.05.2020/06) and registered to Clinical Trials (Registration No: NCT0555534204). The study was conducted under the current Helsinki Declaration. All patients were recorded and informed in the first application to the clinic after their clinical and radiologic examinations. All volunteers signed the informed consent form.

### Study sample

Patients, aged ≥18 years, without any systemic disease, who had asymptomatic, bilateral, similarly positioned impacted third molar that had been indicated for extraction for orthodontic reasons—i.e., Class 1 or Class 2 according to the Pell and Gregory Classification, vertical or mesioangular according to Winter’s Classification, and showed complete mucosal and partial bone retention—were included in this study. Pregnants, breastfeeders, smokers, and patients who failed to attend postoperative follow-up visits, took additional medications, had allergies, and experienced unusual complications were excluded from the study.

### Study variables

The patients were categorized into two groups in terms of suture material to be used, and the PET suture (Ti-cron, Medtronic-Covidien, UK, 3/0, 75 cm, 3/8, reverse cutting and coated with silicon for antibacterial adhesion) was used in Group 1, and the silk suture (Silk, Doğsan, TÜRKİYE, 3/0, 75 cm, 3/8, reverse cutting) in Group 2. Ensuring the double-blinded study design, suturing was performed by an independent surgeon outside the study. In addition, the sutures were soaked in rifamycin solution by the auxiliary staff to avoid distinguishing their color and then transferred to the oral cavity.

### Surgical procedure

**Fig. 1:**
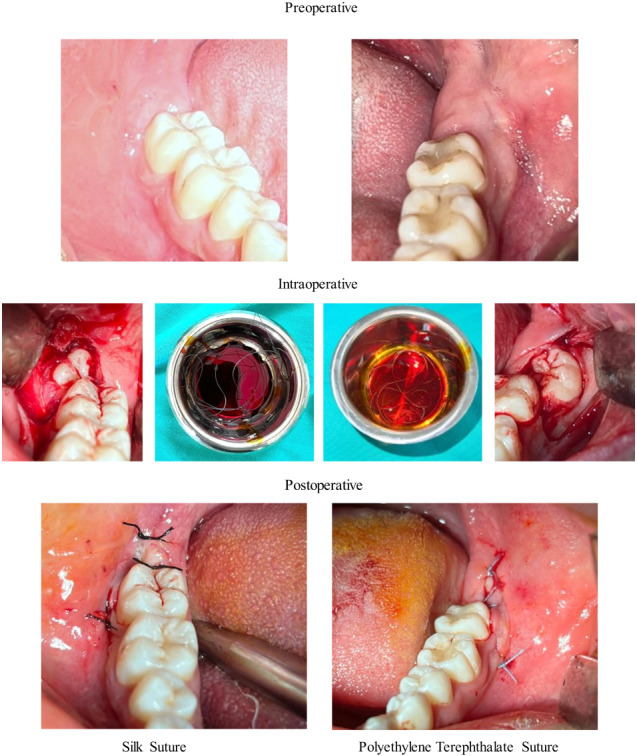
The operations were completed with PET or silk sutures

The same research surgeon performed all surgical protocols. After administering local anesthesia (2-ml articaine hydrochloride 40 mg/ml with epinephrine 0.01 mg/ml, Maxicaine Fort, VEM Drug, İstanbul, Turkey), the area was exposed with a 3-cornered flap. Extractions were completed via bone removal (teeth were separated into pieces when necessary) under saline cooling. The extraction sockets were subsequently irrigated with saline, and bleeding was taken under control. The operations were completed through the primary suture of the wound edges with PET or silk sutures (Fig. 1). Three simple knots were applied along the incision line; one in the vertical corner, one in the horizontal corner, and one in the horizontal distal region. All patients were given ibuprofen (Brufen 600 mg film tablet, 2*1) as analgesic and benzydamine hydrochloride with chlorhexidine gluconate mouthwash (Andorex mouthwash, 200 ML, 3*1) as antiseptic. All sutures were removed on the seventh postoperative day.

### Data collection

**Fig. 2:**
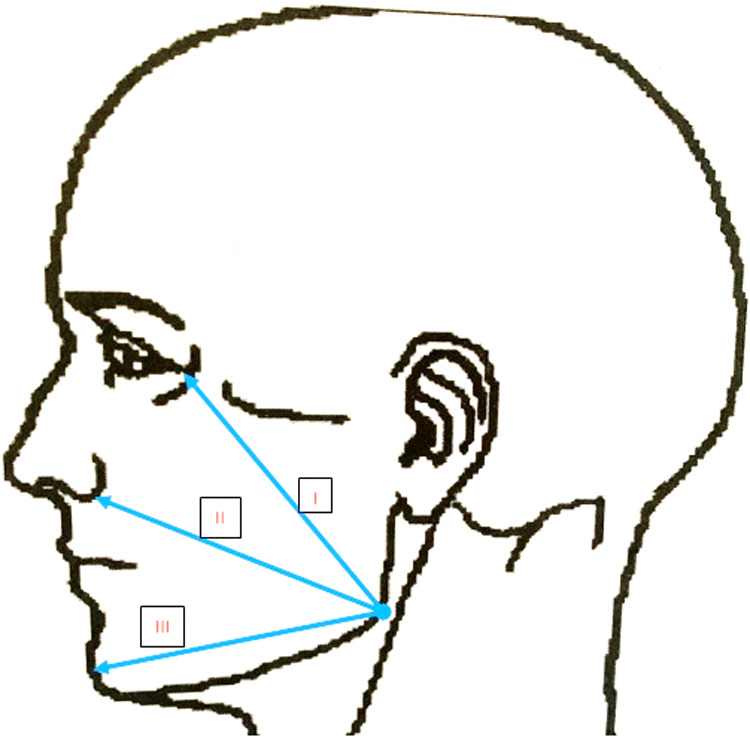
Distances of swelling measurements

**Fig. 3:**
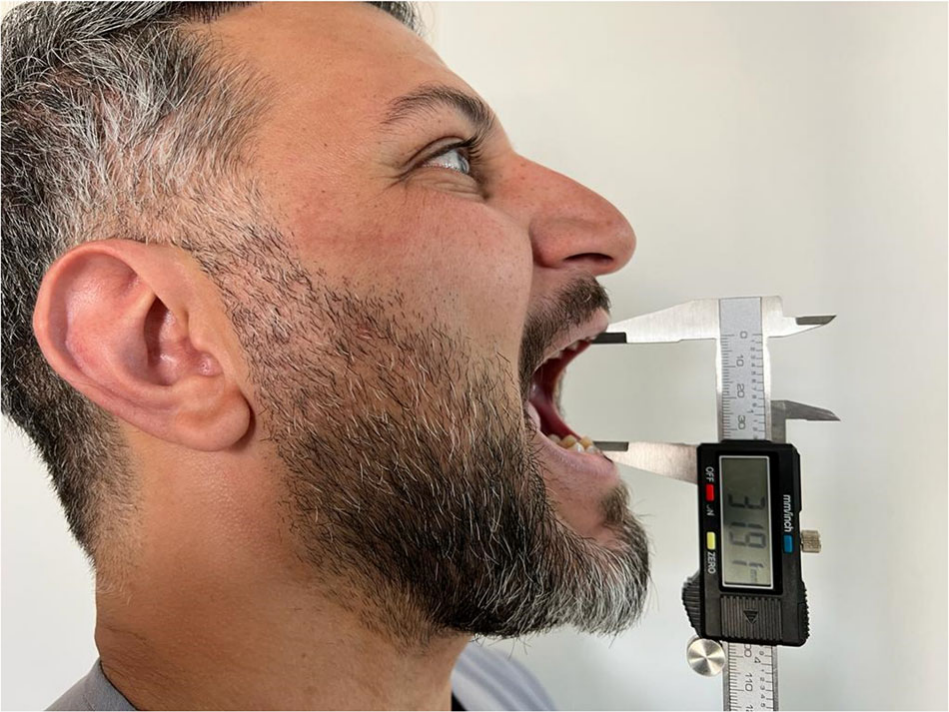
Mouth-opening measurement

The pain score was evaluated via the Visual Analog Scale (VAS) at the postoperative 3rd, 6th, 12th, and 24th hours and on the postoperative 2nd, 3rd, 4th, 5th, 6th, and 7th days. To evaluate the level of pain, patients were instructed to rate it on 10-cm VAS wherein 0 indicated no pain and 10 indicated the worst pain imaginable. For the evaluation of swelling, the distances between the angulus and lateral canthus, angulus and lateral nasal wall, and angulus and pogonion were measured preoperatively with an elastic mm ruler (Fig. 2). Trismus evaluation was performed by preoperatively measuring the distance between the upper and lower central incisors at preoperatively with a digital caliper (Fig. 3). Preoperative measurements were repeated on the postoperative second and 7th days. Plaque accumulation on the suture was examined with visual assessment scoring as 0 indicates no plaque, 1 indicates a small amount of plaque, and 2 indicates plenty of plaque on the postoperative second and 7th days.

### Statistical analysis

According to the power analysis results with 95% confidence (1–α), 80% test power (1–β), *d* = 0.676 effect size, it was calculated that at least 28 patients were required in the study. In the beginning, 57 patients were included in the date range during which the study was conducted. However, seven patients were excluded from the study owing to failure to attend their follow-up visits, five because of alveolitis, two because of infection, and three because of different drug use, and data of the remaining 40 patients were evaluated at the end of the study which was enough according to the power analysis. Choices related to whether the right or left side of the patients would be operated on first and which suture material would be used were randomly determined using online software (http://graphpad.com/quickcalcs/randomize1.cfm).

The data were analyzed with SPSS version 23 program (IBM, Armonk, NY, USA). Compliance with normal distribution was examined via the Shapiro–Wilk Test. The Wilcoxon Test was used to compare dependent data that were not normally distributed in paired groups. The Friedman Test was used to compare non-normally distributed dependent data in groups of three or more, and multiple comparisons were made with Dunn’s Test. The results of the analyses were presented in the form of frequency (percentage) for categorical variables, mean ± standard deviation, and median (minimum-maximum) for quantitative variables. The significance level was taken as *p* < 0.05.

## Results

Of the 40 patients participating in the study, 47.5% (*n* = 19) were male and 52.5% (*n* = 21) were female with a mean of 26.1 ± 7.25 years. The distribution of gender, age, lenght, and weight of the patients included in the study is given in the table (Table 1).

**Table 1:** Frequency distributions and descriptive statistics of demographic characteristics

	Frequency (*n*)	Percentage (%)
Gender		
Male	19	47.5
Female	21	52.5
	Mean ± sd	(min-max)
Age (year)	26.1 ± 7.25	(17–49)
Length (mm)	167.35 ± 8.35	(145–187)
Weight (kg)	65.03 ± 11.02	(45–87)

For the evaluation of trismus, analysis of the differences in mouth-opening values according to preoperative (T0), postoperative 2nd day (T1) and postoperative 7th day (T2) revealed no significant intergroup differences (*p* > 0.05). A statistically insignificant decrease was observed in both groups on T1. However, the comparison of mouth-opening values in the silk (*p* = 0.000) and PET (*p* = 0.000) groups showed that T2 was significantly higher than T1 (*p* < 0.001) (Table 2).

**Table 2:** Comparison of mouth opening values within and between groups according to time

	Silk		PET		p*
	Mean ± sd	(min-max)	Mean ± sd	(min-max)	
Mouth opening T0	**45.28** **±** **7.34**	(32–60)^a^	**45.8** **±** **6.79**	(33–60)^a^	**0.801**
Mouth opening T1	35.13 ± 10.54	(12–55)^b^	36.63 ± 10.63	(11–55)^b^	0.516
Mouth opening T2	**43.28** **±** **8.33**	(21–60)^a^	**43.38** **±** **7.86**	(23–60)^a^	**0.934**
p**	**<0.001**		**<0.001**		

*PET* polyethylene terephthalate

*Wilcoxon Test

**Friedman Test

^a-b^There is no difference between the mouth-opening values with the same letter in each group

Values shown in bold are statistically significant (*p* < 0.001)

When the differences in surface area measurement values for swelling evaluation were analyzed according to preoperative (T0), postoperative 2nd day (T1) and postoperative 7th day (T2), there was no significant difference among groups (*p* > 0.05) (Table 3).

**Table 3:** Comparison of surface area measurement values between groups according to time

	Silk		PET		p*
	Mean ± sd	(min-max)	Mean ± sd	(min-max)	
Swelling1 T1-T0	3.58 ± 6.32	(0–30)	3.53 ± 4.44	(0–15)	0.763
Swelling1 T1-T2	2.9 ± 4.62	(0–20)	3.25 ± 4.3	(0–15)	0.706
Swelling1 T2-T0	0.68 ± 2.32	(0–10)	0.28 ± 0.93	(0–5)	0.477
Swelling2 T1-T0	3.83 ± 5.01	(−10–20)	2.73 ± 3.27	(0–10)	0.205
Swelling2 T1-T2	3.78 ± 4	(0–20)	2.65 ± 3.17	(0–10)	0.257
Swelling2 T2-T0	0.05 ± 2.79	(−15–5)	0.08 ± 0.35	(0–2)	0.524
Swelling3 T1-T0	3.23 ± 4.9	(−15–15)	3.45 ± 4.61	(0–20)	0.836
Swelling3 T1-T2	3.18 ± 3.67	(−5–10)	3.23 ± 4.14	(0–15)	0.824
Swelling3 T2-T0	0.05 ± 1.99	(−10–5)	0.23 ± 0.89	(0–5)	0.854

*Swelling1* distance between angulus and lateral canthus, *Swelling2* distance between the angulus and the lateral wall of the nose, *Swelling3* distance from angulus to pogonion, *T0* initial measurements, *T1* measurements made on the 2nd postoperative day, *T3* measurements made on the 7th postoperative day

*Wilcoxon Test

When VAS values were compared according to time, VAS values for the silk group were higher at the 12th (*p* = 0.011) and 24th hours (*p* = 0.042) (*p* < 0.05). Therefore, the VAS values were significantly higher in the silk group than PET group at the 3rd and 6th hours (*p* = 0.000, *p* < 0.001), whereas the VAS value at the 3rd hour was higher in the PET group (*p* = 0.000, *p* < 0.001) (Table 4).

**Table 4:** Intragroup and intergroup comparisons of VAS values

	Silk		PET		p*
	Mean ± sd	(min-max)	Mean ± sd	(min-max)	
VAS 3rd hour	**4.85** **±** **2.43**	(0–10)^ed^	4.23 ± 2.7	(0–10)^c^	0.154
VAS 6th hour	**4.93** **±** **2.13**	(1–10)^d^	3.95 ± 2.39	(0–10)^c^	0.052
VAS 12th hour	3.9 ± 2.35	(0–8)^cd^	2.55 ± 2.37	(0–9)^c^	**0.011**
VAS 24th hour	3.23 ± 2.3	(0–8)^cd^	2.13 ± 2.37	(0–8)^bc^	**0.042**
VAS 2nd day	2.85 ± 2.52	(0–10)^c^	2.33 ± 2.41	(0–8)^bc^	0.372
VAS 3rd day	2.33 ± 2.52	(0–9)^bc^	1.3 ± 1.73	(0–7)^ab^	0.073
VAS 4th day	1.18 ± 1.99	(0–9)^ab^	0.83 ± 1.52	(0–7)^ab^	0.590
VAS 5th day	1.08 ± 2.21	(0–10)^ab^	0.48 ± 1.36	(0–7)^a^	0.127
VAS 6th day	0.58 ± 1.75	0 (0–9)^a^	0.43 ± 1.53	0 (0–9)^a^	0.720
VAS 7th day	0.8 ± 2.2	0 (0–10)^a^	0.43 ± 1.66	0 (0–10)^a^	0.345
p**	**<0.001**		**<0.001**		

*VAS* visual analog scale, *PET* polyethylene terephthalate

*Wilcoxon Test

**Friedman Test

^a-e^There is no difference between VAS values in each group with the same letter

Values shown in bold are statistically significant (*p* < 0.001)

Values of plaque accumulation on the suture were compared between the groups, and it was observed that the plaque value in the silk group was significantly higher than that in the PET group on the second postoperative day (*p* = 0.005, *p* < 0.05). Also, the value for the silk group on the 2nd day was significantly higher than on the 7th day (*p* = 0.000, *p* < 0.01) (Table 5).

**Table 5:** Intragroup and intergroup comparisons of the presence of plaque on the suture

	Presence of Plaque	Silk		PET			
		*n*	%	*n*	%	P*	P**
Postop 2nd day	0	20	50	30	75	0.005*	0.018
	1	16	40	9	22.5		
	2	14	10	1	2.5		
Postop 7th day	0	32	80	34	85	0.366	0.641
	1	8	20	4	10		
	2	0	0	2	5		

*PET* polyethylene terephthalate

*Wilcoxon Test

**Friedman Test (*P* < 0.05)

## Discussion

PET fibers are used not only as sutures but also in producing many surgical materials. PET is non-absorbable, braided, flexible, and has a rough surface, and it is used as a suture material in orthopedic, ophthalmic, and cardiovascular surgery (11). PET’s biocompatibility, high tension resistance, high tensile and knot strength, and low tissue reaction are other features that support preferable usage in surgery [9]. Since the surface of the PET suture is prone to bacterial adhesion, coatings are applied to improve the surface properties accordingly. The PET suture we used in this study had a silicone coating [12]. Few clinical studies have examined the efficacy of sutures. Therefore, suture selection based on physical and chemical properties may not meet expectations for clinical applications [13]. When selecting an appropriate suture for the operation, choosing sutures with proven clinical performance would be recommended [14, 15]. Naleway et al. and Stankevicius et al. reported that in vivo conditions, knot regions in suture materials reduce the tensile strength of the suture and that this is directly proportional to the time spent in vivo environment [16, 17]. Stankevicius et al. reported that in vivo conditions, knot sites pose a risk for suture breakage, and surgeons should consider this issue clinically [17]. Muftuoglu et al. revealed that silk suture loses its tensile strength by 8% in vivo, and Karaca also revealed that silk suture loses its tensile strength in vivo environment [18, 19]. Karaca and Stankevicius et al. showed that the 3/0 PET suture’s tensile strength did not decrease, increased in contrast under in vivo conditions [17, 19]. In the present split-mouth study, 3/0 silk and PET sutures were compared, and less pain was observed at 12 and 24 h on the PET suture side. We think that the high tensile strength of the PET suture may be associated with less pain.

In studies evaluating the bacterial retention of silk sutures, Sortino et al. showed that aerobic bacterial retention in silk sutures was higher than in polyglycolic acid sutures [20]. Sala-Perez et al. showed that aerobic and anaerobic bacterial retention in silk sutures was higher than in polyglecaprone [21]. Bucci et al. examined the plaque retention of silk, polyamide, and polyglycolide sutures after extraction of impacted wisdom teeth and found that the silk suture had the highest plaque retention [22]. Lekens et al. compared the tissue reaction of silk, ePTFE, a polyester suture after respective periodontal surgery, and found that silk suture caused more extensive tissue reaction with more plaque accumulation [23]. Hafiz et al. reported that the pain felt on the first, third, and seventh postoperative days was higher in the silk group than in the polyglactin group for lower third molar surgery. They also reported that swelling (on the first, third, and seventh postoperative days), redness (on the third and seventh postoperative days), secondary infection (on the postoperative 1st and 3d days), and wound dehiscence were more common with silk sutures [24]. In our study, the effects of silk and PET sutures on swelling were similar. However, more plaque retention was observed in the silk suture on the second postoperative day. The silk suture was thought to cause more plaque retention in the early stages of wound healing, increasing inflammation in the surgical site, and the increased inflammation may be associated with more pain on the side in the 12th and 24th postoperative hours [25, 26]. In addition, we noticed that all alveolitis or infections occurred on the side where silk sutures were used, and these patients were excluded from the study. All these results suggested that silk suture is a risk factor for infection as well as inflammation [20, 21]. When the structural characteristics of silk sutures were evaluated in clinical and in vivo conditions, it was shown that silk sutures cause more inflammation, plaque retention, bacterial involvement, and infection compared to other sutures [27]. Therefore, the use of silk sutures in impacted third molar surgery and other oral surgical procedures should be questioned regarding their clinical utility [28]. In the present study, sutures were removed the seventh postoperative day. If the sutures had been kept in the oral environment for a longer time, different results could have been obtained for the two sutures at times to be evaluated.

The limitation of this study, the structures and contents of the nutrients consumed by individuals after both impacted tooth operations could not be standardized, which may have affected postoperative plaque retention.

## Conclusion

In conclusion, plaque accumulation in the PET suture was significantly lower on the postoperative 2nd day, and patients felt significantly lower pain at the 12th and 24th hours. According to our study, these results support that PET sutures might be a more comfortable option instead of silk sutures in impacted third molar surgery.
